# Aftermath of the Tuberculosis Congress

**Published:** 1910-03-12

**Authors:** 


### SPECIAL ARTICLES.

### AFTERMATH OF THE TUBERCULOSIS CONGRESS.

By A SPECIAL WASHINGTON CORRESPONDENT.

Now that a year has elapsed, it is easy to see that in Washington at least the Tuberculosis Congress has made a profound impression upon a number of people. To some of these it has appealed in a practical way. For instance, the lady principal of one of the high schools of Columbia now sleeps in a tent-bed on an open balcony.

The minds of people in general are better prepared for the recommendations urged by sanitarians. Prompted by your correspondent, and instigated by the Chairman of the Board of Charities, of whom I shall have more to say, the street-car employés have been again directed to keep open one set of ventilators at least in each car. It remains to be seen whether this essential safeguard will be preserved even now, when the weather becomes so severe as to make the inevitable draught more uncomfortable. So far it has been generally done. Another important precaution in connection with the cars requires to be taken. I allude to the keeping the rear door closed, for while it is open the stopping of the car causes a back draught, and this raises a thick cloud of dust from the platform and the street. The smooth asphalt of the streets dries within an hour or two of a rainfall, so that the dust is not held to the ground as it is in the interstices of the blocks of the old granite sett roads, nor can it adhere as it does to the rather greasy surface of wooden paved streets such as London now possesses. The consequence is that even a short time after rain the cars raise a cloud of fine dust; this necessarily contains numerous bacteria, some of which have in other cities been shown to be pathogenic. Besides this, the material of which it is composed dries up the secretions of the mucous membranes, and must much interfere with the moist filtration action of the nares, and permit the passage of many organisms which would otherwise have been arrested by a surface normally moist. Short of abolishing the partially closed platform with which the cars are at present constructed, it will be necessary to flush down the floor probably twice daily, and in addition to keep the rear door closed during the process of stopping the car; this will be a difficult matter unless exit is arranged for through the front door.

One can safely say that any such recommendations would have been ignored by the tramway companies had they been mooted before the Tuberculosis Congress, for when a special committee of the Inter-state Commerce Commission was charged last summer with the special supervision of the street railways of Washington, considerable recalcitrance was displayed by the companies. The Commission, however, is now in working order, and the companies appear to be trying to conform to any recommendation they consider reasonable; and as they are considerably influenced by public sentiment, the function of the Congress in shaping this has been a most important one with regard to the sanitation of cars.

The windows of the residents are much more often kept open now than formerly. This is still done in most cases in an unintelligent way, for they rarely open them from the top, and what ventilation does occur from below is more or less hampered by thick curtains. But even this signifies the desire to practise the lessons conveyed by the Congress. Moreover, when one recommends a different manner or greater amount of ventilation, one is no longer met with the assertion that it is perfect, or the look which questions one's sanity, or even the polite acquiesence of indifference. It is more usual to meet with an acquiescence of intelligent interest or an apologetic adoption of one's recommendations.

Churches and their meeting halls are notorious for bad ventilation; and it was very gratifying to note that when the Welsh Choir sang in a local church here, the pastor himself, before the performance began, requested the audience to open the windows. Theatres, too, leave much to be desired in this respect; and your correspondent has often asked the management to improve the ventilation during a performance. Since the Congress an immediate response has been the rule.

It must be said, however, that in these respects other large cities are less backward than Washington. Their progress has been greatly due to the efforts of the medical profession, who in the instances of Chicago, Baltimore, and San Francisco have co-operated with the newspapers to secure the wide dissemination of authentic information such as the care of the person, hygiene of the dwelling, choice of food, safeguarding against infection, and so on. In this connection a movement is now on foot for securing the performance of a similar office through the agency of the medical profession of Washington by means of official pronouncements by the Medical Society of the District of Columbia. This organisation is the second oldest in the United States, and has conservative traditions: but nevertheless it last year lent itself to the exploitation of a movement organised by one or two medical men and others against the impurity of the milk supply of the city.

In Chicago, this work is being done chiefly by the Department of Health. Its officers are endowed with much more power than the public health officers in England, as they themselves are executive officers, and have at their disposal a detail of police with which to enforce their regulations. Its present Chief Commissioner is responsible for the additional means now employed there in the crusade against disease; for he has instituted a system of public lectures in churches, schools, clubs, mothers' meetings, etc., and these average about one a day. In Washington a similar work has been carried on, especially in the schools, by the Tuberculosis Committee, a small body of doctors and laymen which has now transformed itself into the Anti-tuberculosis Association, whose membership is open on payment of one dollar. The scope of its work is similar to that of the organisations which have so long existed in Great Britain. The committee had already helped in the founding of a tuberculosis dispensary, now in operation about four years, a tuberculosis sanatorium, opened last autumn, and a country sanitarium, a private corporation receiving people of moderate means. Each of these is at present on only a small scale; but it is to be hoped that the late Congress will afford the stimulus and means for their rapid growth.

The most encouraging movement instigated by the Congress is the display of the exhibits in other cities. It has already been shown almost entire in Philadelphia and New York. In the latter over 800,000 persons visited it, and these did not come altogether out of idle curiosity; for demonstrations and lectures were largely attended all day long. "Feature" days were instituted; and on one of these over 60,000 Hebrews attended. On another day, the Italians, and upon other days other nationalities were assembled; lectures were given in their several languages, and each of the various types of school was taken over the exhibit. There were days for business men, club women, physicians, and natives of different States; and in this and other ways the exhibit was brought to public notice, and people stimulated to the initiative.

The public interest in tuberculosis is reflected even in the advertising pages of the newspapers. The advertisement of a well-known whisky is carefully concealed in the midst of a half column advertisement, emphasising tuberculosis as the scourge of mankind, and that it may be prevented, even though there is no positive cure. It states that a million lives annually can be saved by its prevention, gives praise to the men whose work increases our power over it, quotes Secretary Cortelyou's statement that 160,000 persons died of tuberculosis in the United States last year. Appeals for an organisation under authority of law for the prevention and cure of tuberculosis in every city and village in the country, and that these should provide accommodation for the treatment of cases, and possess powers and separate curable cases from incurable ones. It then speaks of the hygiene, rest, and good food required, and then states "no treatment or measures for the cure of this disease can be as successful as one reinforced by a refined and wholesome stimulant like this medicinal whisky." Further instructions are then given, and the name and address of the seller concludes this remarkable advertisement.

What is likely to have a most important consequence is the entering into the movement of the order of Christian Brothers, who have issued a circular letter of instruction to the superiors of all the educational institutions in the province comprising the middle southern States. These include colleges, academies, protectory and industrial and parish schools. Moreover, aspirants to the order are to give particular attention to the study of "methods preventive of tuberculosis in so far as they relate to the protection of the young."

